# Normal and disordered gastric emptying in diabetes: recent insights into (patho)physiology, management and impact on glycaemic control

**DOI:** 10.1007/s00125-022-05796-1

**Published:** 2022-10-04

**Authors:** Ryan J. Jalleh, Karen L. Jones, Christopher K. Rayner, Chinmay S. Marathe, Tongzhi Wu, Michael Horowitz

**Affiliations:** 1 Endocrine and Metabolic Unit, Royal Adelaide Hospital, Adelaide, Australia; 2 Adelaide Medical School, The University of Adelaide, Adelaide, Australia; 3 Centre of Research Excellence in Translating Nutritional Science to Good Health, The University of Adelaide, Adelaide, Australia; 4 Department of Gastroenterology and Hepatology, Royal Adelaide Hospital, Adelaide, Australia

**Keywords:** Gastric emptying, Gastroparesis, Glucagon-like peptide-1, Hyperglycaemia, Hypoglycaemia, Incretin, Review

## Abstract

Gastric emptying is a major determinant of postprandial blood glucose, accounting for ~35% of variance in peak glucose in both healthy individuals and those with type 2 diabetes. Gastric emptying is frequently disordered in individuals with diabetes (both abnormally delayed and accelerated). Delayed gastric emptying, i.e. diabetic gastroparesis, may be linked to upper gastrointestinal symptoms for which current treatment remains suboptimal; pharmacological acceleration of delayed emptying is only weakly associated with symptom improvement. Accordingly, the relationship between symptoms and delayed gastric emptying is not simply ‘cause and effect’. In insulin-treated patients, disordered gastric emptying, even when not associated with gastrointestinal symptoms, can cause a mismatch between the onset of insulin action and the availability of absorbed carbohydrate, leading to suboptimal glycaemic control. In patients with type 2 diabetes, interventions that slow gastric emptying, e.g. glucagon-like peptide-1 receptor agonists, reduce postprandial blood glucose. This review focuses on recent insights into the impact of gastric emptying on postprandial blood glucose, effects of diabetes therapy on gastric emptying and the management of disordered gastric emptying in diabetes. In view of the broad relevance of gastric emptying to diabetes management, it is important that future clinical trials evaluating novel therapies that may affect gastric emptying should quantify the latter with an appropriate technique, such as scintigraphy or a stable isotope breath test.

**Graphical abstract:**

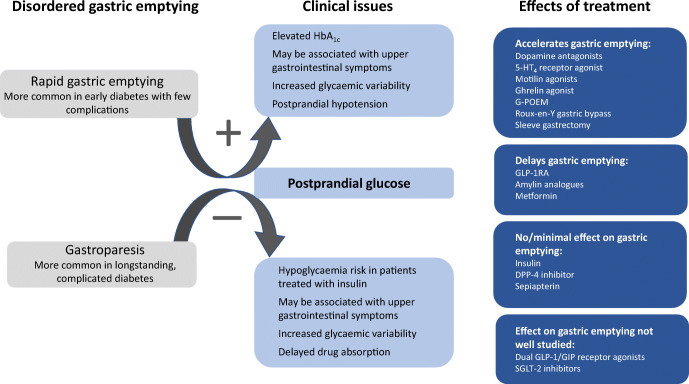

**Supplementary Information:**

The online version of this article (10.1007/s00125-022-05796-1) contains a slideset of the figures for download, which is available to authorised users.

## Introduction

*‘There is nothing like looking, if you want to find something… You certainly usually find something, if you look, but it is not always quite the something you were after.’*

J.R.R. Tolkien, The Hobbit

Gastric emptying is now appreciated to be central to the pathophysiology and rational management of diabetes (type 1, type 2 and pancreatogenic). This relatively recent recognition represents a paradigm shift where the outcomes of methodologically sound research in humans, with and without diabetes, have in many cases refuted long-established dogma. Gastroparesis is defined by abnormally delayed gastric emptying in the absence of mechanical obstruction. Once thought to be relatively rare, a belief influenced by the outcome of epidemiological studies that defined gastroparesis based on upper gastrointestinal symptoms without measuring gastric emptying, it is now recognised that gastric emptying is abnormally slow in 30–50% of individuals with long-standing, complicated, type 1 or type 2 diabetes [1–3]. It is also appreciated that gastroparesis has clinical implications beyond that of symptoms, including malnutrition, glycaemic instability and impaired absorption of orally administered drugs. By contrast, in uncomplicated type 2 diabetes, gastric emptying is often accelerated [4, 5]. Acute changes in blood glucose affect gastric emptying, which is slowed during hyperglycaemia and accelerated during hypoglycaemia [6]. Recently, it has been recognised that gastric emptying, even when normal, is a major, and hitherto generally underappreciated, determinant of the magnitude of the postprandial rise in blood glucose, which, in many individuals with diabetes, predominates over fasting blood glucose in determining average glycaemic control as assessed by HbA_1c_. Accordingly, gastric emptying represents a specific therapeutic target [6, 7]. In the broadest sense, the action of glucose-lowering therapy, particularly insulin, should ideally be coordinated closely with the rate of delivery of dietary carbohydrate and subsequent absorption from the small intestine. This review focuses on these advances in knowledge, including their implications for more personalised and effective management of diabetes.

## Pathophysiology of diabetic gastroparesis

**Fig. 1:**
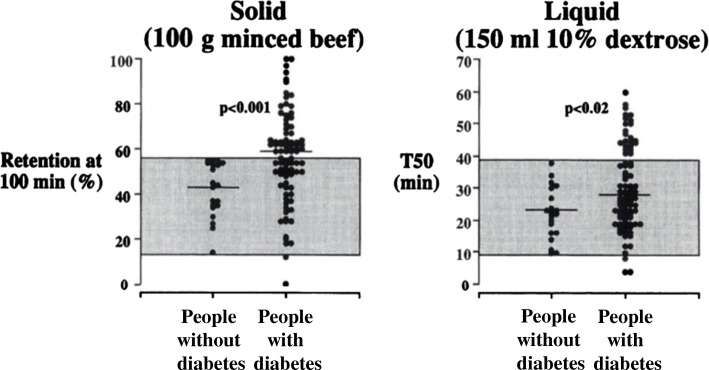
Gastric emptying of solid (100 g minced beef, per cent retention at 100 min) and liquid (150 ml 10% dextrose, half-emptying time [T50]) nutrients in 86 participants with long-standing diabetes (66 with type 1 diabetes, 20 with type 2 diabetes) and 20 healthy participants. Horizontal lines represent median values. The range of gastric emptying rates in the healthy participants is represented by the shaded area. Adapted from Jones et al [8] © SNMMI. This figure is available as part of a downloadable slideset

**Fig. 2:**
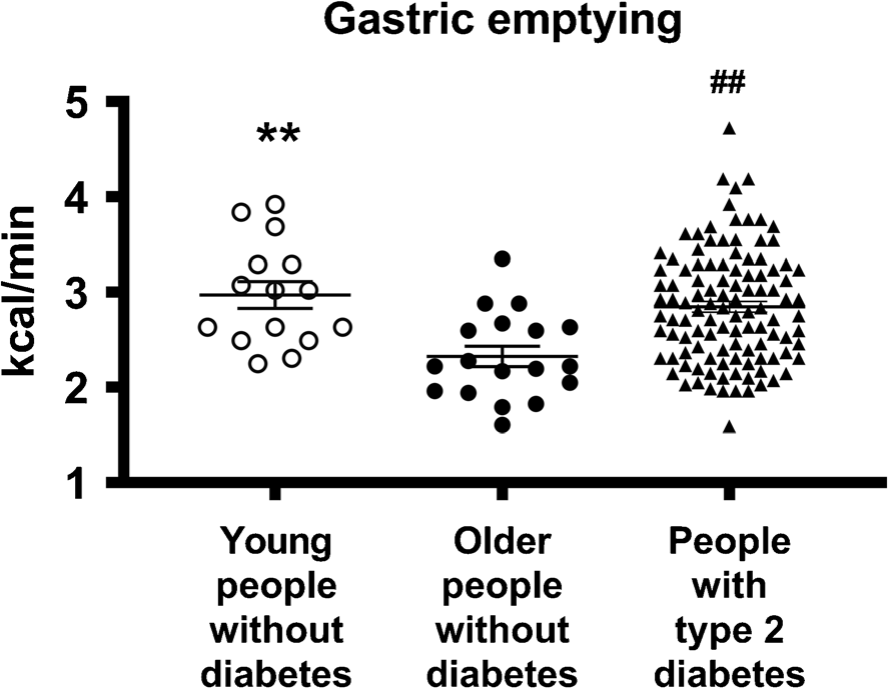
Gastric emptying of a mashed potato meal in people with type 2 diabetes (*n*=111), and in young (*n*=15) and older (*n*=18) control participants without diabetes, as assessed by ^13^C-octanoic acid breath test and expressed as kcal/min. **p<0.01 for young vs older participants without diabetes, ^##^*p*<0.01 for people with type 2 diabetes vs older control participants without diabetes. Unpaired *t* test used to determine statistical difference. Adapted from Watson et al [4] by permission of Oxford University Press on behalf of the Endocrine Society. © The Endocrine Society. This figure is available as part of a downloadable slideset

In people with and without diabetes, gastric emptying of solid and liquid nutrients exhibits a wide inter-individual, but much lesser intra-individual, variation [1, 2, 4] (Fig. 1). While predominantly a pulsatile, rather than continuous, phenomenon, gastric emptying of most nutrients approximates an overall linear pattern (in the case of solids following an initial lag phase of 20–30 min), ranging between 4 and 17 kJ/min (1–4 kcal/min) in healthy individuals [6, 8]. Accordingly, in many individuals, whether healthy or with diabetes, a 75 g oral glucose load (~1255 kJ), as in an OGTT, will not have emptied completely from the stomach at 120 min (as this would require an emptying rate ≥ 11 kJ/min) [7] (Fig. 2). In some ethnic populations (Han Chinese, Mexican Americans and American Indians) predisposed to type 2 diabetes, gastric emptying appears to be more rapid than in individuals of European descent [9, 10].

Within the enteric nervous system, the interstitial cells of Cajal (ICC) act as pacemakers and, accordingly, play a major role in control of gastrointestinal motor function [6, 11]. It is not widely appreciated, however, that in healthy individuals, gastric emptying of nutrients is regulated primarily by inhibitory neurohormonal feedback arising from their interaction with the small intestine, rather than ‘intragastric’ mechanisms, and that both the length and region of small intestine exposed to nutrients modulate this feedback [12, 13]. Recent studies by the Gastroparesis Clinical Research Consortium (GCRC) in the USA, where full thickness gastric biopsies have been obtained in individuals with refractory gastroparesis, have yielded important insights relating to the pathophysiology of diabetic gastroparesis [11]. The latter is now recognised to be both complex and heterogeneous [6]. While there are abnormalities in vagal innervation, contrary to expectation, the relationship of delayed gastric emptying with autonomic dysfunction, as assessed by cardiovascular reflex tests, is weak [1, 2]. Loss or damage to the ICCs appears to be a dominant abnormality [3, 6, 11], where altered immune function with a shift from M2 to M1 macrophage expression and impaired regulation of haem oxygenase 1 leading to increased oxidative stress may be responsible [6, 11]. Other common abnormalities include reductions in intrinsic nerves and inhibitory neurons expressing nitric oxide synthase (NOS) [3, 6]. Not surprisingly, gastroduodenal motor and sensory dysfunctions in gastroparesis are also highly variable; frequent abnormalities include impaired relaxation of the proximal stomach, reduced antral contractility and abnormalities of the gastric electrical rhythm [3, 6].

## Evaluation of gastric emptying

In the evaluation of suspected disordered gastric emptying, drugs that affect it should be withheld where possible and upper gastrointestinal endoscopy should be performed routinely to exclude mechanical obstruction [6, 14]. Scintigraphy remains the ‘gold-standard’ technique for quantifying gastric emptying and allows measurement of both solid and liquid emptying, potentially concurrently, although frequently only solid emptying is assessed. Current guidelines recommend a test meal of eggs, toast, jam and water, with only a solid component (eggs) radiolabelled [14, 15]. It should, however, be appreciated that the relationship of solid and liquid emptying is relatively weak [1]. Recommendations to standardise the methodology for scintigraphy include ensuring blood glucose concentrations are <15 mmol/l during measurement [15]. Limitations of scintigraphy include the need for expensive, specialised equipment and exposure to radiation. A more recently developed, and acceptable, alternative to scintigraphy is the stable isotope breath test, which is now well-validated, simple to perform and avoids exposure to ionising radiation [16]. Moreover, isotope breath test data can be adjusted, using the so-called ‘Wagner-Nelson’ method, to yield values comparable to scintigraphy [16]. The paracetamol absorption test (i.e. evaluation of the plasma kinetics of an oral paracetamol dose) is, unfortunately, still used widely to measure gastric emptying, particularly by the pharmaceutical industry. However, this technique has substantial limitations including uncertainty as to which component of a meal the paracetamol is emptying with, and the inability to measure gastric emptying of solids [17, 18]. In our opinion, this method should not be used in isolation for either clinical or research purposes, particularly given the availability of the isotope breath test. Similar considerations apply to the widespread assessment of gastrointestinal symptoms using participant self-report, which is known to be unreliable, rather than validated questionnaires, which are now mandated by regulatory authorities in studies of drug therapy for functional gastrointestinal diseases [19]. Evaluation of gastric emptying using the wireless motility capsule is a newer technique; however, the indigestible capsule tends to be emptied later than nutrient liquids and solids, and it may be insensitive for detecting abnormally rapid gastric emptying [3, 6]. Other newer techniques for measuring gastric emptying, including MRI and three-dimensional ultrasound, remain primarily in the research domain [3, 6].

## Impact of gastric emptying on blood glucose

Gastric emptying is a major determinant of postprandial blood glucose in people with or without diabetes, accounting for ~35% of the variance in peak glucose, and both the timing and significance of this relationship are impacted by glucose tolerance status [6]. In healthy individuals, relatively more rapid gastric emptying is associated with a greater glycaemic response in a 75 g OGTT at 30 min, but not 60 min, and the relationship is inverse at 120 min, presumably reflecting effective glucose counter-regulation [7]. In contrast, in individuals with impaired glucose tolerance (IGT), relatively more rapid gastric emptying is associated with a greater glycaemic response at 30 min and 60 min, but not 120 min. In individuals with type 2 diabetes, there is a further ‘shift to the right’ so that the glycaemic response is related directly to gastric emptying both at 60 min and 120 min [7, 20]. These observations have implications for use of the OGTT to diagnose type 2 diabetes, IGT (i.e. 120 min plasma glucose) and gestational diabetes. An understanding of the determinants of the blood glucose at 60 min may be the strongest predictor of future type 2 diabetes [21]. Given the relationship between blood glucose and gastric emptying, the use of continuous blood glucose monitoring has the potential to predict disordered gastric emptying. It should also be appreciated that, in contrast to long-standing, complicated type 1 or type 2 diabetes, where gastric emptying is frequently delayed [1–3], type 2 diabetes of short duration and uncomplicated type 1 diabetes in adolescents are both associated with abnormally rapid gastric emptying [5, 22]. In type 1 diabetes, it has been suggested that this acceleration reflects the reduction in human islet amyloid pancreatic peptide secretion [23]. Older individuals with type 2 diabetes tend to have slower gastric emptying compared with the young, consistent with the modest slowing of gastric emptying observed with healthy ageing [4]. Rapid gastric emptying may also be associated with upper gastrointestinal symptoms, which cannot be discriminated from those associated with gastroparesis [24].

**Fig. 3:**
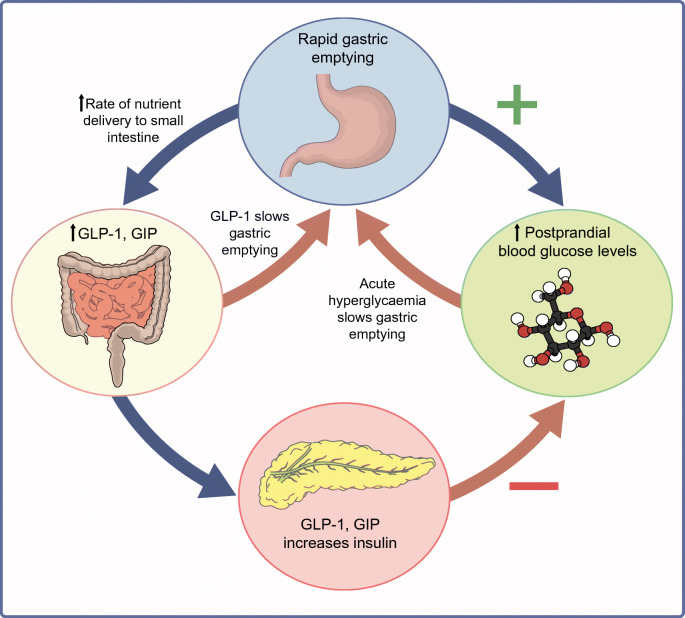
Schema of the interdependent relationships of gastric emptying, incretin hormones and blood glucose. Gastric emptying is both a determinant of, and determined by, blood glucose. This figure is available as part of a downloadable slideset

In many instances, the magnitude of the delay in gastric emptying in individuals with diabetes and gastroparesis is modest [1, 2]. The rate of gastric emptying also tends to remain relatively stable for up to 25 years [25]. Acute changes in blood glucose affect gastric emptying; hyperglycaemia induced by i.v. glucose slows emptying, although there is recent evidence that this may not apply to spontaneous elevations in blood glucose [26]. In contrast to hyperglycaemia, acute insulin-induced hypoglycaemia (blood glucose ~2.0–2.6 mmol/l) robustly accelerates gastric emptying in individuals with type 1 diabetes, even in those with cardiovascular autonomic neuropathy and gastroparesis [27] (Fig. 3). This is likely to represent an important counterregulatory mechanism and warrants further study [27].

In individuals with type 1 diabetes or type 2 diabetes who are managed with insulin, ‘carbohydrate counting’ is widely used to determine insulin type and dosage; however, this approach fails to account for the substantial variation between individuals in the rate of delivery of carbohydrate to the small intestine. Accordingly, the choice and dosage of rapid-acting insulin should be considered carefully in individuals with disordered gastric emptying to optimise compatibility with the altered rate of absorption of carbohydrate. For example, if an ultra-rapid-acting insulin tends to cause postprandial hypoglycaemia, an insulin with a slower onset of action may avoid this. In this context, gastric emptying should have major implications (which remain to be evaluated) for algorithms relating to the optimal use of insulin pumps linked to real time subcutaneous glucose monitoring systems in type 1 diabetes.

Because gastric emptying regulates the entry of nutrients into the small intestine, it modulates the secretion of the two incretin hormones, glucose dependent insulinotropic polypeptide (GIP) and glucagon-like peptide-1 (GLP-1) [28]. The insulinotropic effect of GIP, the dominant incretin in healthy individuals, is markedly attenuated in type 2 diabetes, whereas that of GLP-1 is relatively preserved [28]. Experimental models have demonstrated a prompt, and load-related, stimulation of GIP in response to direct intraduodenal infusion of glucose [6]. In contrast, there is minimal stimulation of GLP-1 when glucose is infused intraduodenally at rates of 4–8 kJ/min (1–2 kcal/min), but a substantial response at 13–17 kJ/min (3–4 kcal/min) in both healthy individuals and those with type 2 diabetes [12, 29]. It has recently been shown that there is a direct relationship between the rate of gastric emptying of oral glucose and the stimulation of plasma GLP-1 in response to intraduodenal glucose [30], indicating that the rate of gastric emptying in each individual is modulated by the GLP-1 response to small intestinal nutrients. Studies using a specific antagonist of GLP-1, exendin 9-39, have demonstrated that endogenous GLP-1 slows gastric emptying in healthy individuals [31].

There is little information about the impact of gastric emptying on blood glucose in the large number of individuals with pancreatogenic diabetes. In the most studied form of pancreatogenic diabetes, cystic fibrosis-related diabetes (CFRD), small intestinal inhibitory feedback from the products of fat digestion is attenuated as a result of pancreatic exocrine insufficiency. Consequently, gastric emptying is often abnormally rapid, and the secretion of both GLP-1 and GIP is attenuated, favouring an increase in the postprandial glycaemic excursion [32]. Management of exocrine pancreatic insufficiency and associated malabsorption with enzyme supplementation slows gastric emptying and augments the incretin response, reducing postprandial glucose [32]. In patients who have undergone partial pancreatectomy, particularly proximal resection, there is often a transient post-operative delay in gastric emptying, which may result in a reduction in the glycaemic response to an OGTT [33].

## Effect of diabetes therapies on gastric emptying

A number of drugs used in the management of diabetes slow gastric emptying, which contributes to their capacity to reduce postprandial blood glucose.

### GLP-1 receptor agonists

GLP-1 receptor agonists (GLP-1RAs) are used widely in the management of type 2 diabetes, and increasingly in obesity, and can be classified as either ‘short-’ or ‘long-acting’ based on their plasma half-lives. The short-acting GLP-1RAs, exenatide twice daily (BD) [34] and lixisenatide [35–38], have a robust, albeit variable, effect to slow gastric emptying. The magnitude of this deceleration is dependent on the baseline rate of gastric emptying (i.e. individuals with gastroparesis may potentially not exhibit further slowing, although this remains to be determined) and is predictive of the magnitude of postprandial glucose lowering [34]. The implication is that improvement in glycaemic control by short-acting GLP-1RAs, as assessed by HbA_1c_ in type 2 diabetes, may be dependent on the baseline rate of gastric emptying, particularly in individuals in whom fasting blood glucose is relatively well controlled. The effects of long-acting GLP-1RAs (i.e. exenatide once weekly [QW], liraglutide, dulaglutide, semaglutide [subcutaneous and oral] and tirzepatide [a dual GIP/GLP-1 receptor agonist]) on gastric emptying have, for the main part, been evaluated with the suboptimal paracetamol test [17, 39–43], rather than a more accurate technique [44]. It has also been suggested that long-acting GLP-1RAs do not have an effect to slow gastric emptying with longer-term use because of tachyphylaxis induced by sustained exposure of GLP-1 receptors. However, in humans, the slowing of gastric emptying by i.v. GLP-1 is diminished over 24h, but the effect is still significant [45]. While the slowing of gastric emptying appears to be less with long-acting GLP-1RAs compared with short-acting GLP-1RAs, substantial and durable slowing of gastric emptying clearly persists with both liraglutide [37, 46] and exenatide QW [44], two long-acting GLP-1RAs for which gastric emptying has been quantified using a methodologically sound technique. In the latter case, the reduction in postprandial glucose has been shown to be related to the extent of this delay [44]. Accordingly, it appears that both short- and long-acting GLP-1RAs slow gastric emptying (Table 1). Moreover, the European Medicines Agency considers that delayed gastric emptying is an adverse effect of liraglutide [47]. What remain to be clarified are the magnitude, duration and dose-relationship of these effects—issues that can only be resolved if gastric emptying is measured in clinical trials using an appropriate method. Of note, studies that have shown that slowing of gastric emptying reduces postprandial blood glucose have generally not discriminated effects on gastric emptying from those on small intestinal motility/transit. The potential for GLP-1RAs to inhibit small intestinal transit may represent a substantial contribution to postprandial glucose lowering [48].

**Table 1:** Effects of GLP-1RAs on gastric emptying in humans

GLP-1RA	Dose and duration	Health condition	Method of gastric emptying measurement	Effect on gastric emptying
Short-acting				
Exenatide BD				
Drucker et al [42]	5 μg sc BD for 14 weeks	T2D (*n*=50)	Paracetamol absorption	Exenatide vs placebo: +
Linnebjerg et al [34]	5 or 10 μg sc BD for 5 days	T2D (*n*=7)	Scintigraphy	Exenatide vs placebo: +++
Lixisenatide				
Jones et al [35]	10 μg sc daily (single dose)	T2D (*n*=15) Healthy individuals (*n*=15)	Scintigraphy	Baseline vs lixisenatide: +++ (in health and T2D)
Lorenz et al [38]	5 μg sc daily initially, increased by 2.5 μg every 5 days up to 20 μg sc daily (total duration 28 days)	T2D (*n*=43)	Stable isotope breath test	Baseline vs day 28 lixisenatide: +++
Rayner et al [36]	5 μg sc daily initially, increased to 10 μg sc daily after 7 days and then 20 μg sc daily after 14 days (total duration 56 days)	T2D (*n*=30)	Scintigraphy	Baseline vs day 56 lixisenatide: +++
Long-acting				
Dulaglutide				
Barrington et al [43]	5 and 8 mg sc weekly for 5 weeks	T2D (*n*=15)	Paracetamol absorption	Dulaglutide vs placebo: ++
Exenatide QW				
Drucker et al [42]	2 mg sc weekly for 14 weeks	T2D (*n*=24)	Paracetamol absorption	Exenatide vs placebo: –
Jones et al [44]	2 mg sc weekly for 8 weeks	Obesity (*n*=32)	Scintigraphy	Exenatide vs placebo: +
Liraglutide				
Halawi et al [46]	3 mg sc daily for 16 weeks	Obesity (*n*=40)	Scintigraphy	Liraglutide vs placebo: ++
Meier et al [37]	0.6 mg sc daily initially, increasing by 0.6 mg sc each week to either 1.2 or 1.8 mg sc daily (total duration 8 weeks)	T2D (*n*=94)	Stable isotope breath test	Baseline vs week 8: liraglutide: ++
Semaglutide (subcutaneous)				
Hjerpsted et al [40]	0.25 mg sc weekly for 4 weeks, then 0.5 mg for 4 weeks, then 1 mg sc weekly (total duration 12 weeks)	Obesity (*n*=30)	Paracetamol absorption	Semaglutide vs placebo: ++
Semaglutide (oral)				
Dahl et al [39]	3 mg daily for 4 weeks, then 7 mg daily for 4 weeks, then 14 mg daily for 3 days	T2D (*n*=15)	Paracetamol absorption	Semaglutide vs placebo: ++
Tirzepatide (Dual GLP-1 and GIP receptor agonist)				
Urva et al [41]	Tirzepatide 0.5, 1.5, 4.5, 5, 10 or 15 mg sc weekly (total duration 4 weeks)	T2D (*n*=53) Healthy individuals (*n*=33)	Paracetamol absorption	Baseline vs week 4 tirzepatide: ++

Only the most relevant studies are included

sc subcutaneous; T2D, type 2 diabetes; –, no effect on GE; +, gastric emptying delayed by <25% compared with baseline/placebo; ++, gastric emptying delayed by 25–50% compared with baseline/placebo; +++, gastric emptying delayed by >50% compared with baseline/placebo

The insights from such studies are likely to facilitate much more personalised use of GLP-1RAs and have a number of other implications, including the timing of cessation of long-acting GLP-1RAs prior to endoscopy or surgery, where there are recent anecdotal reports of retained gastric contents despite ‘appropriate’ periods of fasting [49].

The effect of GLP-1RAs on gastric emptying may also be of relevance to their potential use in type 1 diabetes. In addition to suppressing glucagon, the slowing of gastric emptying may attenuate the counterregulatory acceleration of gastric emptying by hypoglycaemia [50], which could increase the risk of hypoglycaemia, as observed in a trial with liraglutide [51]. Because gastric emptying was not measured concurrently in this trial [51], this potential mechanism cannot be confirmed. While it has been suggested that the frequent gastrointestinal adverse effects (which may compromise longer-term usage) and appetite suppression induced by GLP-1RAs are related to slowing of gastric emptying, this may not be the case [52]. Another potential example of ‘targeted’ use of GLP-1RAs is CFRD, where abnormally rapid gastric emptying can be slowed using the short-acting GLP-1RA exenatide BD, to reduce the postprandial glycaemic excursion [53].

A substantial (>20mmHg) fall in blood pressure after meals (postprandial hypotension) occurs in up to 40% of individuals with long-standing, complicated diabetes (more common than orthostatic hypotension), and is associated with more rapid gastric emptying [35]. The slowing of gastric emptying by GLP-1RAs may attenuate the postprandial fall in blood pressure and could prove useful in the management of postprandial hypotension, which currently lacks an effective treatment [35].

### DPP-4 inhibitors

In contrast to GLP-1RAs, dipeptidyl peptidase-4 (DPP-4) inhibitors have minimal, if any, effect on gastric emptying in type 2 diabetes, although their postprandial glucose lowering may be greater in individuals in whom gastric emptying is relatively faster [54]. The absence of an effect on gastric emptying is likely to reflect the much lesser stimulation of GLP-1 receptors than with GLP-1RAs; moreover, DPP-4 inhibition prevents the conversion of inactive PYY1-36 to the active 3-36 form which slows gastric emptying [55].

### Other glucose-lowering medications

Metformin, when delivered intraduodenally, slows gastric emptying modestly in type 2 diabetes in the absence of nausea [56]. The mechanism(s) may reflect greater stimulation of GLP-1 from the distal gut resulting from a reduction in proximal intestinal glucose absorption, inhibition of bile acid resorption, and/or alterations in the gut microbiota [56]. Pramlintide, an amylin analogue, slows gastric emptying substantially, which contributes to its marked effect to lower postprandial glucose [6]. Acarbose, an alpha-glucosidase inhibitor that delays carbohydrate absorption, may increase exposure of the L cells in the distal gut to nutrients, enhancing GLP-1 secretion. In some experimental paradigms, acarbose has been shown to slow gastric emptying, although this was not evident with a mixed meal in type 2 diabetes [57]. While, to our knowledge, the effect of sodium–glucose cotransporter 2 (SGLT2) inhibitors on gastric emptying has not been evaluated, there is no reason to anticipate a significant effect. Lastly, while insulin may influence gastric emptying through modulating blood glucose levels, hyperinsulinaemia per se does not appear to affect gastric emptying [58].

### Effects of weight loss procedures for obesity on gastric/pouch emptying

Gastric emptying of nutrient liquids is markedly accelerated following Roux-en-Y gastric bypass [59] and sleeve gastrectomy [60], leading to a supraphysiologic GLP-1 response that attenuates the rise in blood glucose and, in some cases, subsequent hypoglycaemia [61]. Gastric emptying of solids following gastric bypass may be delayed or accelerated; emptying is influenced by concurrently ingested liquids [62] and may relate to weight loss [63].

Fluid-filled (but not air-filled) intragastric balloons slow gastric emptying of solids substantially [64], probably because air-filled balloons float to the fundus of the stomach, whereas fluid-filled balloons reside in the antrum and impair grinding and emptying of solids.

## Management of gastroparesis associated with upper gastrointestinal symptoms

**Fig. 4:**
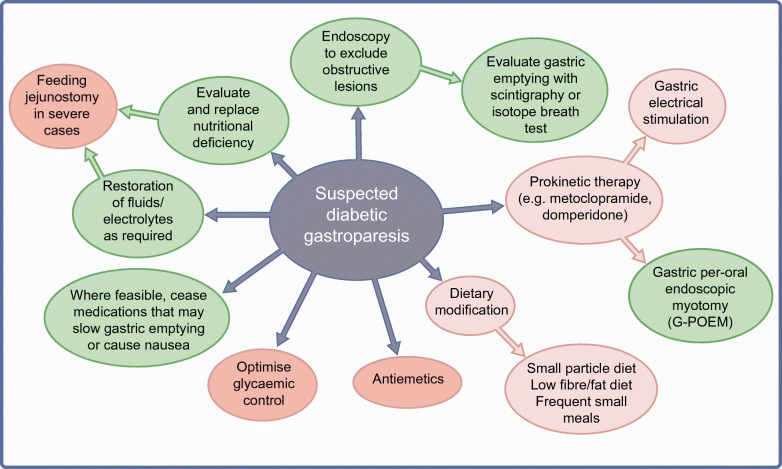
Recommended management of suspected diabetic gastroparesis. Green, standard clinical practice/approaches supported by controlled trials; light pink, approaches supported by limited evidence; dark pink, approaches that may have a role but remain to be evaluated adequately. This figure is available as part of a downloadable slideset

Although it represents a substantial health burden, treatments for symptomatic gastroparesis remain limited and suboptimal (Fig. 4). Progress has been compromised by two long-standing assumptions which have proved to be incorrect: (1) that delay in gastric emptying is the cause of symptoms, rather than being a marker of underlying dysfunction of gastroduodenal control mechanisms; and (2) that making the stomach pump more effectively would inevitably lead to symptom improvement. A further limitation is that much of the current evidence is derived from poorly designed trials. For these reasons, while the heterogeneous nature of the motor/sensory dysfunctions in diabetic gastroparesis has implications for the development of more effective therapy, treatment should be targeted primarily at the relief of specific, bothersome symptoms.

### Non-pharmacological management

Dietary recommendations, e.g. consumption of frequent, small meals and avoidance of high fat foods, while intuitively logical, remain to be evaluated adequately [65]. Numerous drugs slow gastric emptying, but ceasing them may not prove feasible. Optimising glycaemic control is often recommended; in addition to slowing gastric emptying, acute hyperglycaemia may be associated with increased upper gastrointestinal ‘sensitivity’ [6]. However, this is often challenging and the rationale for the approach is hitherto only supported by uncontrolled studies. The effect of improvement in chronic glycaemic control (HbA_1c_) remains uncertain with an absence of controlled studies, although a prospective study reported normalisation of delayed gastric emptying, measured with scintigraphy, following improved glycaemic control [66]. A small trial in individuals with type 1 diabetes and gastroparesis found that hybrid closed loop insulin pump therapy improved the mean percentage time that blood glucose was in range (3.9–10 mmol/l) from 26% to 58.4%, however neither gastrointestinal symptoms nor gastric emptying were evaluated [67]. Nutritional support (e.g. feeding into the small intestine) may be necessary when there are severe, refractory symptoms associated with dehydration or malnutrition, but again, the evidence base for this approach is limited [65].

### Pharmacological management

Pharmacological treatment has focused on prokinetic medications, but, as discussed, it is now appreciated that the relationship between symptomatic improvement and acceleration of gastric emptying is, at best, modest [68].

### Dopamine receptor antagonists

Metoclopramide, a dopamine-2 (D2) receptor antagonist, is the only pharmacologic treatment currently approved by the US Food and Drug Administration (FDA) for diabetic gastroparesis, but it has not been approved for this indication by the European Medicines Agency (EMA). Oral metoclopramide has been used for decades, but recently a nasal formulation, which may be absorbed more reliably, has been shown to accelerate gastric emptying and provide symptomatic relief in women (but not men) with diabetic gastroparesis [69]. Based on anecdotal reports, subcutaneous metoclopramide may be effective in treating vomiting episodes. Other D2 receptor antagonists, including domperidone, may improve symptoms to a comparable extent to metoclopramide [70]. Tardive dyskinesia (more common with metoclopramide than domperidone) and QTc (corrected QT interval) prolongation with the potential for arrhythmia are important concerns, although the risk of tardive dyskinesia may be as low as 0.1% per 1000 patient-years with metoclopramide [71]. Nonetheless, metoclopramide carries a black box warning that chronic use (>12 weeks) may result in irreversible tardive dyskinesia [14]. A newer dopamine D2/D3 receptor antagonist, trazpiroben (TAK-906), developed to have reduced cardiovascular and neurologic adverse effects, failed to show symptom improvement [72].

### 5-HT_4_ receptor agonists

Prucalopride, a selective 5-hydroxytryptamine 4 (5-HT_4_) receptor agonist, was effective in improving symptoms in a cohort with predominantly idiopathic gastroparesis, but despite accelerating gastric emptying in a diabetes-specific group, did not improve symptoms [73]. Velusetrag, a selective 5-HT_4_ receptor agonist, accelerates gastric emptying in diabetic gastroparesis, but effects on symptoms were not evaluated [74]. For other 5-HT_4_ agonists, including clebopride, cinitapride and mosapride, there is relatively weak evidence to support efficacy [73], and cisapride, which appeared to be effective at relieving symptoms, was withdrawn from the market in 2000 due to concerns about cardiovascular safety [73].

### Motilin receptor agonists

The macrolide antibiotics, erythromycin and azithromycin, are also motilin receptor agonists and, at least in the short term, accelerate gastric emptying, although their effect on symptoms is uncertain [73]. There are also concerns regarding potential drug-interactions and a reduction in long-term efficacy due to tachyphylaxis [73]. Mitemcinal, a newer motilin receptor agonist, was reported to improve symptoms in diabetic gastroparesis only in the subgroup of patients with BMI <25 kg/m^2^ and HbA_1c_ <86 mmol/mol (10%), for uncertain reasons [75].

### Other therapies for gastroparesis

Medications used to treat chronic abdominal pain, such as pregabalin and gabapentin, have not been formally evaluated in diabetic gastroparesis. The tricyclic antidepressant nortriptyline failed to improve symptoms in idiopathic gastroparesis [76]. Numerous classes of medication used to treat nausea, including 5-HT_3_ antagonists, phenothiazines, cannabinoids and H1 receptor antagonists such as diphenhydramine, have not been evaluated adequately in the context of diabetic gastroparesis but may have a place in its management [3].

### Novel pharmacological targets

The neurokinin-1 receptor antagonist tradipitant was reported to improve nausea in gastroparesis, but the study was underpowered to show a benefit in the subgroup with diabetes [77]. Sepiapterin, an essential cofactor of NOS, has been reported to improve gastric accommodation, but not symptoms, in women with diabetic gastroparesis [78]. Relamorelin, a ghrelin agonist, led to both symptomatic improvement and acceleration of gastric emptying in three studies, but the subsequent phase 3 trial was terminated prematurely, apparently for commercial reasons [79].

### Endoscopic, surgical and other therapies

A number of invasive therapies have been used in individuals with refractory diabetic gastroparesis, but none can currently be considered to have an established place in management. The choice of therapy is, accordingly, empirical.

Gastric per-oral endoscopic pyloromyotomy (G-POEM) involves tunnelling through the submucosa to cut the pyloric sphincter. In a recent sham-controlled, randomised clinical trial of patients with refractory gastroparesis (41% had diabetes), G-POEM achieved symptomatic improvement in 71% of participants compared with 22% following the sham procedure [80]. Gastric emptying, measured by scintigraphy, was accelerated after G-POEM but there was no relationship of symptom improvement with gastric emptying. In another recent larger but uncontrolled prospective study in gastroparesis [81], at 12 months symptoms had improved in 56% of participants who underwent G-POEM and gastric emptying, measured by scintigraphy, normalised in 47% with a moderate correlation between symptom improvement and acceleration of gastric emptying [81]. These observations suggest that G-POEM may have a place in the management of individuals with refractory symptomatic gastroparesis [80].

The delivery of low energy, high frequency pulses to the antrum (gastric electrical stimulation) is effective in the treatment of vomiting in individuals with and without diabetes, regardless of whether they had gastroparesis, as shown in a randomised, crossover study [82]. Gastric emptying was not accelerated, nor was there an improvement in overall quality of life [82].

Open-label studies initially suggested a benefit of intrapyloric injection of botulinum toxin; however, subsequent sham-controlled trials in gastroparesis of various aetiologies failed to demonstrate improvement in either symptoms or gastric emptying [83]. Likewise, a systematic review of 32 studies failed to support acupuncture for management of symptomatic gastroparesis [84].

There is anecdotal evidence that pancreatic transplantation may be effective in managing diabetic gastroparesis [85]; the effect of islet cell transplantation has not been reported. Roux-en-Y gastric bypass surgery [86], sleeve gastrectomy [87] and gastrojejunostomy [88] have also been reported to improve symptoms in refractory gastroparesis, but in view of the small number of cases and the absence of controlled trials, further evaluation is required.

## Conclusions

The seminal importance of gastric emptying in diabetes is now established and recent insights have impacted clinical practice. Clarification of a number of important, unresolved concepts will be dependent on the outcomes of future targeted research. The relative lack of progress in the management of gastroparesis associated with gastrointestinal symptoms reflects, in part, the pursuit of overly simplistic concepts and the fundamental importance of defining pathophysiology for the rational development of novel and effective therapy. The latter must be evaluated in well-designed clinical trials, which is itself challenging. In relation to postprandial glycaemic control, the clinical relevance of gastric emptying would be defined much more clearly if its measurement (e.g. by breath test) was incorporated widely in clinical trials where postprandial glycaemic excursions represent a major endpoint. Mechanisms should not be ignored when they can be assessed both safely and simply. The words of Tolkien are relevant to our quest to understand diabetic gastroparesis; the latter will inevitably prove elusive unless we look carefully and with an open mind.

## Supplementary information

Slideset of figures (PPTX 756 kb)

## Abbreviations

BD: Twice daily
CFRD: Cystic fibrosis-related diabetes
D2: Dopamine-2
GIP: Glucose dependent insulinotropic polypeptide
GLP-1: Glucagon-like peptide-1
GLP-1RA: Glucagon-like peptide-1 receptor agonist
G-POEM: Gastric per-oral endoscopic pyloromyotomy
5-Hydroxytryptamine: 5-HT
ICC: Interstitial cells of Cajal
IGT: Impaired glucose tolerance
NOS: Nitric oxide synthase
QW: Once weekly
