# Supplementary material for: Normal and disordered gastric emptying in diabetes: recent insights into (patho)physiology, management and impact on glycaemic control
Source: Diabetologia. 2022 Oct 4;65(12):1981–93. doi: 10.1007/s00125-022-05796-1 (PMC9630190; doi:10.1007/s00125-022-05796-1)
Supplement: Supplementary file 1 — (PPTX 756 kb) [file 125_2022_5796_MOESM1_ESM.pptx]

## Slide 1
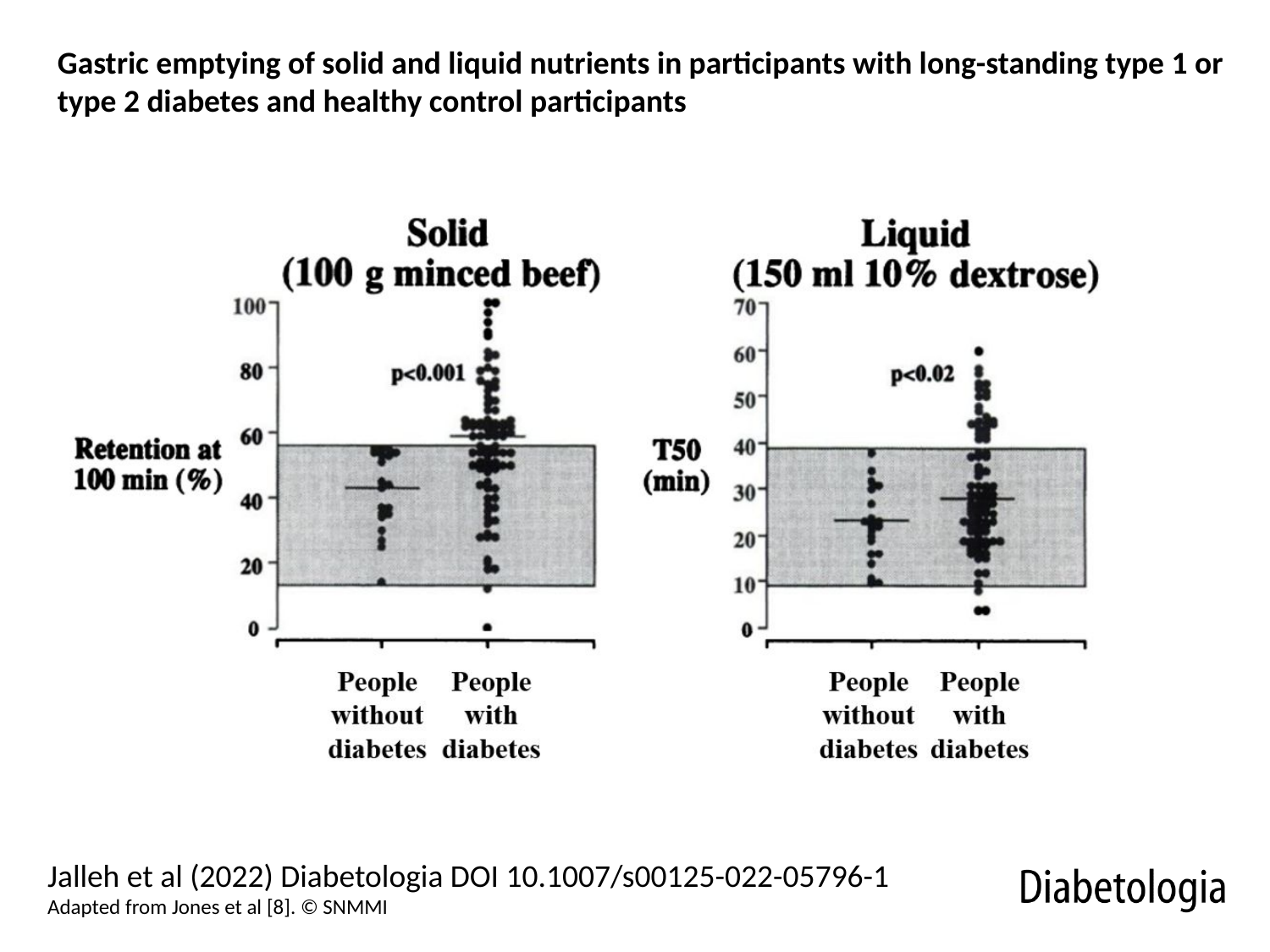

Gastric emptying of solid and liquid nutrients in participants with long-standing type 1 or type 2 diabetes and healthy control participants
Jalleh et al (2022) Diabetologia DOI 10.1007/s00125-022-05796-1
Adapted from Jones et al [8]. © SNMMI

## Slide 2
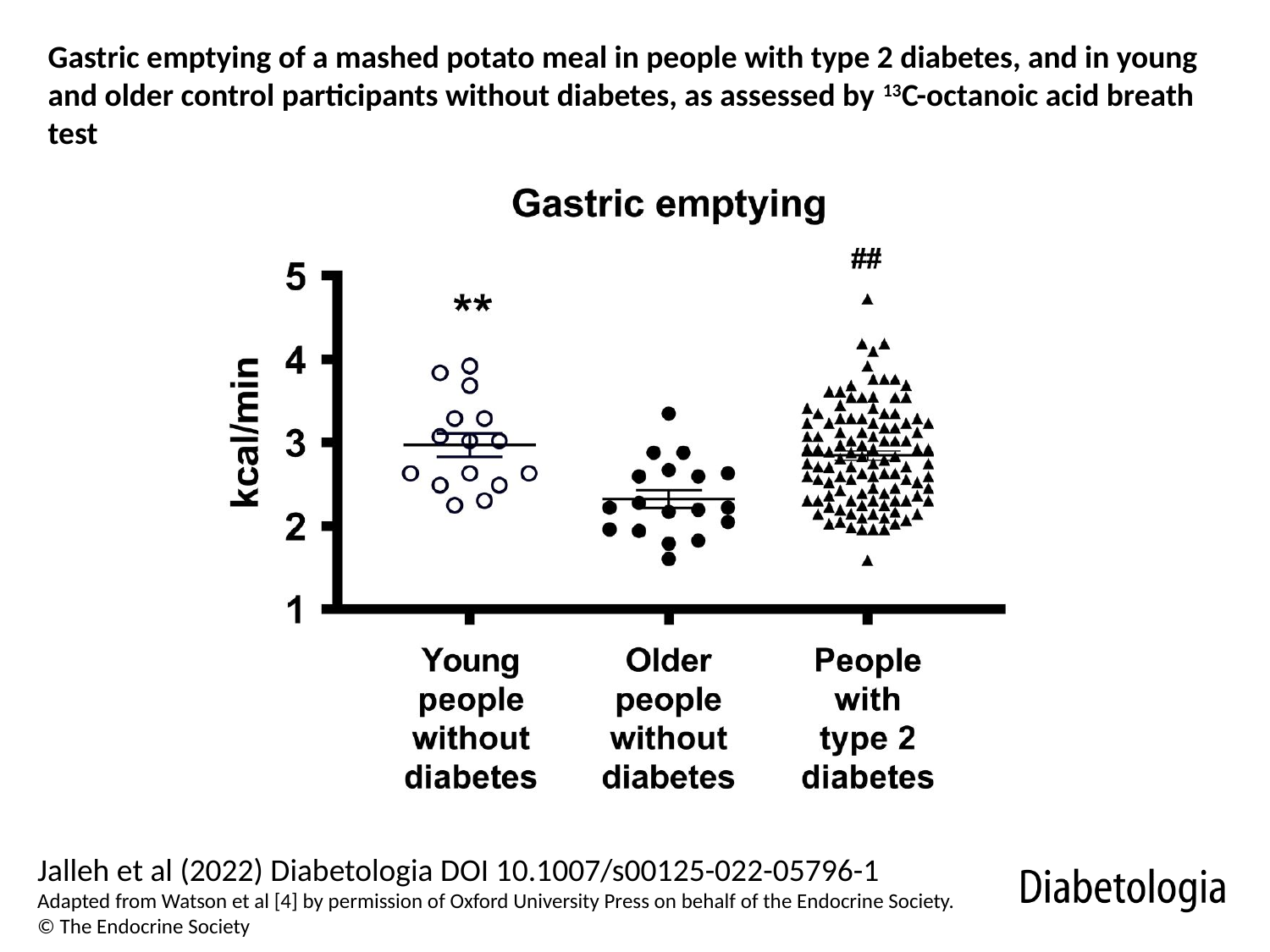

Gastric emptying of a mashed potato meal in people with type 2 diabetes, and in young and older control participants without diabetes, as assessed by 13C-octanoic acid breath test
Jalleh et al (2022) Diabetologia DOI 10.1007/s00125-022-05796-1
Adapted from Watson et al [4] by permission of Oxford University Press on behalf of the Endocrine Society. © The Endocrine Society

## Slide 3
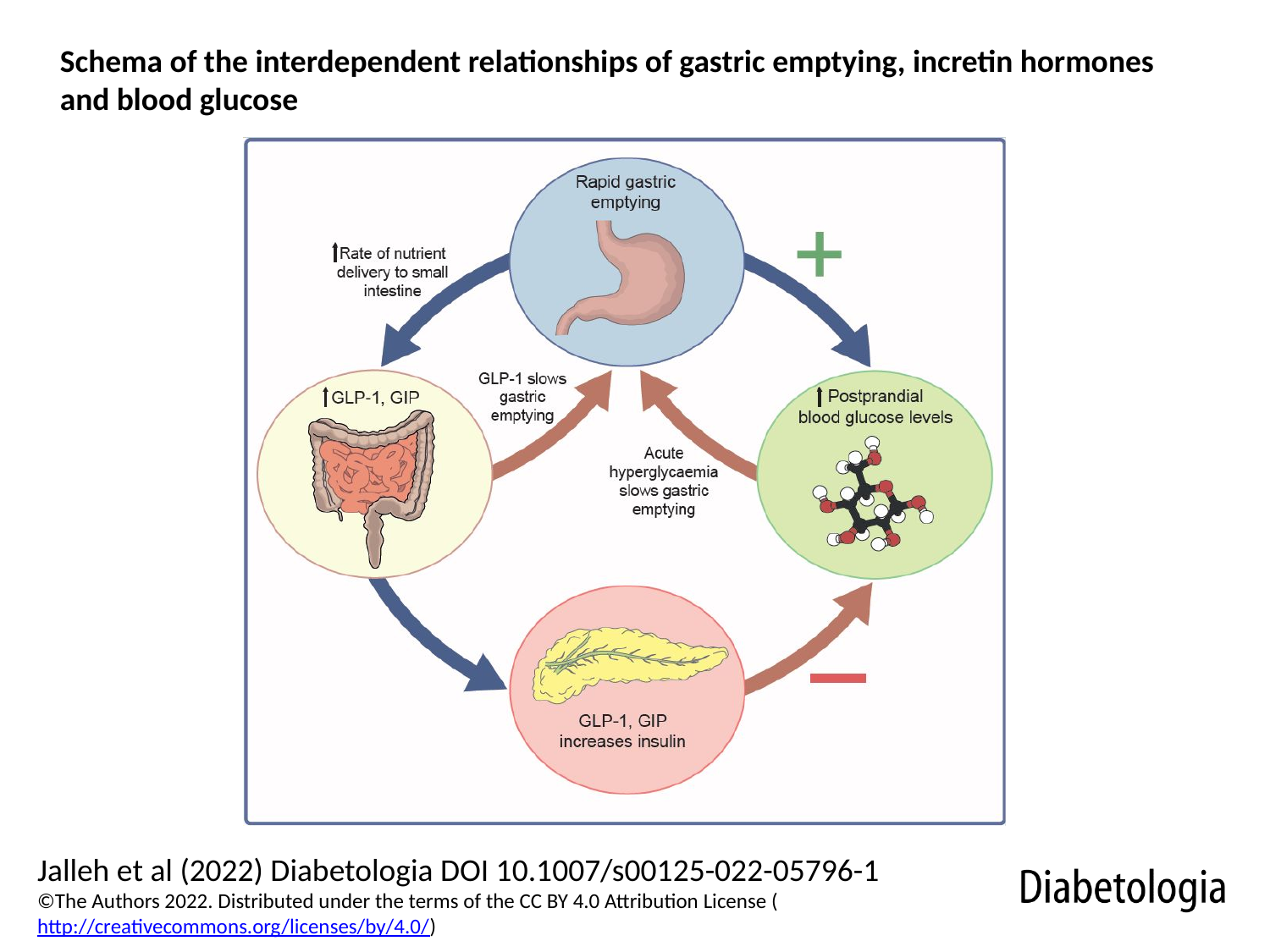

Schema of the interdependent relationships of gastric emptying, incretin hormones and blood glucose
Jalleh et al (2022) Diabetologia DOI 10.1007/s00125-022-05796-1
©The Authors 2022. Distributed under the terms of the CC BY 4.0 Attribution License (http://creativecommons.org/licenses/by/4.0/)

## Slide 4
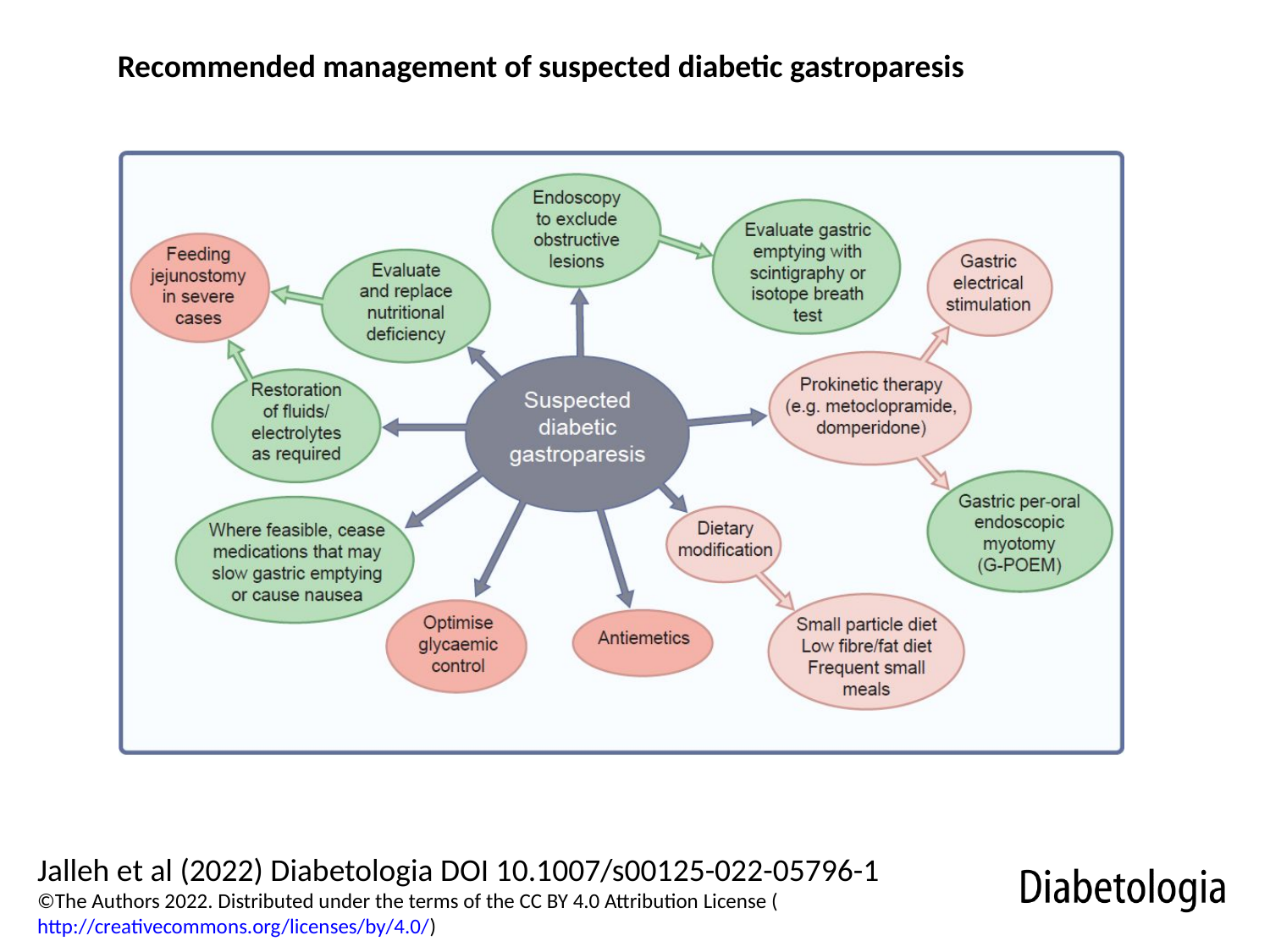

Recommended management of suspected diabetic gastroparesis
Jalleh et al (2022) Diabetologia DOI 10.1007/s00125-022-05796-1
©The Authors 2022. Distributed under the terms of the CC BY 4.0 Attribution License (http://creativecommons.org/licenses/by/4.0/)
